# Persistent Radiculopathy Subsequent to Selective Nerve Root Block

**DOI:** 10.7759/cureus.46468

**Published:** 2023-10-04

**Authors:** Ryan J McLoughlin, Annabelle Jin, Eric A Canlas, Franklin E Caldera, Yejia Zhang

**Affiliations:** 1 Physical Medicine and Rehabilitation, Hospital of the University of Pennsylvania, Philadelphia, USA; 2 Physical Medicine and Rehabilitation, University of Pennsylvania, Philadelphia, USA

**Keywords:** cytotoxicity, compression, magnetic resonance imaging, nerve root block, radiculopathy

## Abstract

Transforaminal selective nerve root blocks are commonly performed for low back pain but are not without risk. This case report describes a 55-year-old man who underwent transforaminal selective nerve root block at the left lumbar (L) 4, L5, and sacral (S) 1 levels for radiating low back pain in the setting of moderate narrowing of the left L4-L5 foramen with impingement on the exiting left L4 nerve roots seen on magnetic resonance imaging (MRI). He developed left foot drop immediately after the procedure and presented to the acupuncture clinic two weeks later with persistent pain, left foot drop, and paresthesia of the left lateral shin. A repeat MRI of the lumbar spine showed mild enhancement of the left cauda equina, including the L5 and possibly L4 nerve roots. The large volume of injection into an area with neuroforaminal narrowing as well as the cytotoxicity of the contrast and anesthetic agents may have contributed to axon damage and left foot drop.

## Introduction

Lumbar radiculopathy occurs when there is injury or harm to the nerve roots as they exit the spine. This condition has the potential to impact individuals across the board and can arise from factors such as disc degeneration, disc herniation, or other forms of trauma [1]. Compression of one or more nerve roots is the most common cause of foot drop and inability to dorsiflex the foot and the toes due to muscle weakness [2]. Transforaminal selective nerve root blocks are procedures routinely performed for lumbar-sacral radiculopathies [3], a condition often marked by pain radiating to the legs. During this procedure, a radio-opaque contrast agent is injected to verify the epidural space, and a blend of medications, including a local anesthetic and steroid is injected into the epidural space where the irritated nerve roots are situated. The local anesthetic disrupts the cycle of pain and spasms, inhibiting the transmission of pain signals within the spinal column. Simultaneously, the steroid helps reduce inflammation.

## Case presentation

The patient is a 55-year-old man with a past medical history of hypertension and hyperlipidemia and a surgical history of lumbar (L) 5/sacral (S)1 microdiscectomy. About 10 years after the successful microdiscectomy, he redeveloped severe back pain radiating anterolaterally down the left lower extremity, associated with numbness. Magnetic resonance imaging (MRI) of the lumbar spine showed disc protrusions at the L4-L5 and L5-S1 levels with moderate narrowing of the left L4-L5 foramen and impingement on the exiting left L4 nerve roots.

**Figure 1:**
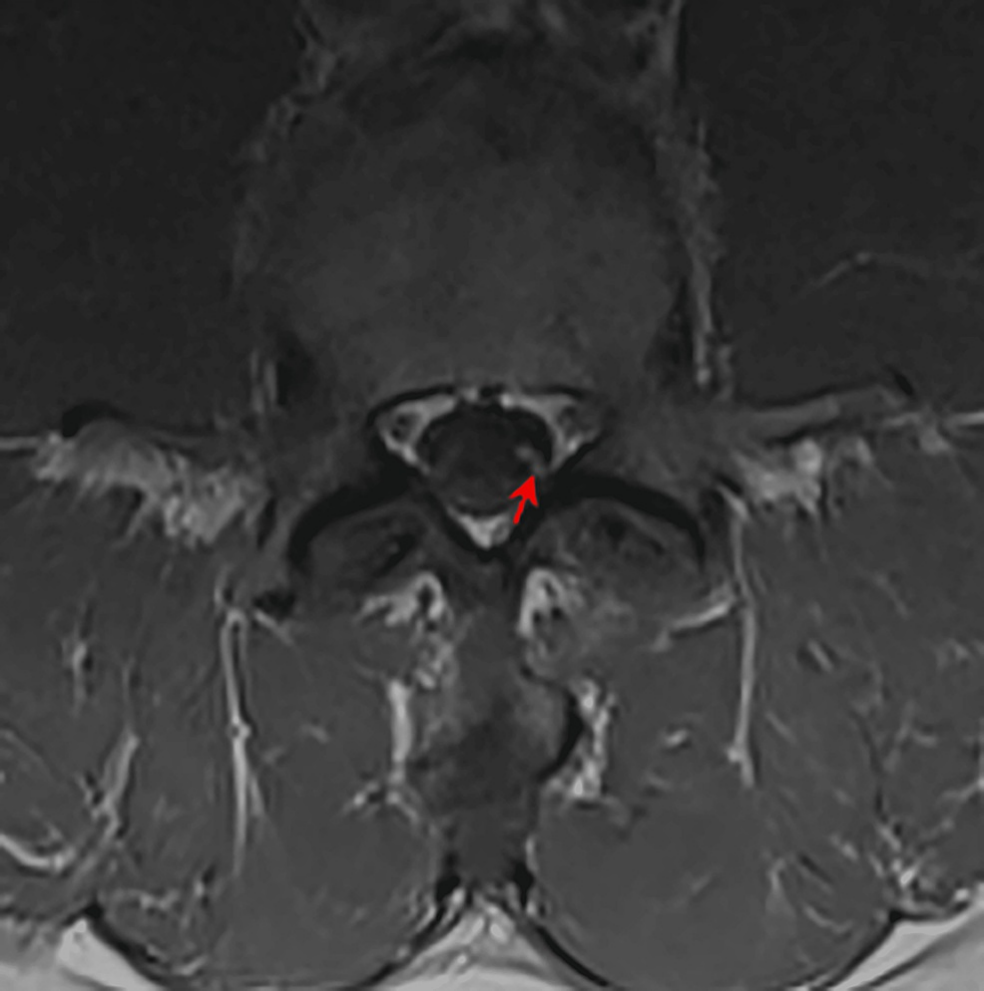
MRI lumbar spine image Arrow: enhanced left L5 nerve root

He underwent transforaminal selective nerve root block at the left L4, L5, and S1 levels. Specifically, 1 milliliter (ml) of iohexol was injected into each neuroforamen to confirm needle placement near the nerve roots, followed by 3 ml of 0.25% bupivacaine plus 40 mg of methylprednisolone acetate, also into each of the three neuroforamina. He developed a foot drop immediately after the procedure and was observed in the clinic for an hour before being discharged home. He presented to the acupuncture clinic two weeks after the injections and complained of worsening back and leg pain and paresthesia of the left lateral shin. Examination showed left ankle dorsiflexor weakness, anterior tibial muscle atrophy, and diminished left patella tendon reflex compared to the right. MRI of the lumbar spine was obtained and showed mild enhancement of the left cauda equina, including the L5 and possibly L4 nerve roots (Figure 1). The MR images were reviewed by a radiologist.

Complete blood count (CBC) showed a normal neutrophil count with a slight increase in monocyte count, which did not suggest any infection. He was given oral methylprednisolone tapering over seven days and underwent physical therapy and acupuncture treatments. At the 18-month follow-up, his left ankle dorsiflexor strength had improved from anti-gravity (3/5) to moderate resistance (4/5) with a corresponding improvement in his gait, although he continued to experience paresthesia of the left lateral shin and reduced left patella tendon reflex compared to the right.

## Discussion

Foot drop has not been reported following selective nerve root injections, although there have been rare occurrences following spinal anesthesia [4]. Differential diagnosis of foot drop after selective nerve root block includes bleeding, infection, and very rarely, neuritis and direct nerve damage by the needles. In the case presented here, MRI suggested L5 and/or L4 radiculopathy without bleeding, and CBC did not suggest any infection. It was unclear if the increase in blood monocyte count was of any clinical significance. The left foot drop, muscle atrophy, and patella tendon hyporeflexia occurred in a patient with preexisting neuroforamen narrowing. He received a higher-than-normal volume of injectables [5], including contrast agents, bupivacaine, and steroid, which are cytotoxic [6-9]. For lumbar selective nerve root blocks, 1 mL of medication and anesthetics are usually injected following up to 0.5 mL of contrast [5]. A “safe” injection volume would depend on the size of the patient’s neuroforamen. The large volume of the injection in this patient (total over 4 ml, with 1 ml of contrast, 3 ml of bupivacaine, and likely 0.5 ml of methylprednisolone) may have caused direct nerve root compression. Even a transient mechanical injury to the nerve root can result in sustained axonal pathology [10]. In addition, rapid injection, compared with slower push, may have caused transiently higher pressure within the neuroforamina.

## Conclusions

In summary, this patient with neuroforamen narrowing due to degenerative spinal changes had additional pressure on the nerve roots from the larger than normal injection volumes. Cytotoxicity of the contrast and anesthetic agents may have contributed to axon damage.

## References

[1] Berry JA, Elia C, Saini HS, Miulli DE (2019). A review of lumbar radiculopathy, diagnosis, and treatment. Cureus.

[2] Liu K, Zhu W, Shi J, Jia L, Shi G, Wang Y, Liu N (2013). Foot drop caused by lumbar degenerative disease: clinical features, prognostic factors of surgical outcome and clinical stage. PLoS One.

[3] Kanaan T, Abusaleh R, Abuasbeh J (2020). The efficacy of therapeutic selective nerve block in treating lumbar radiculopathy and avoiding surgery. J Pain Res.

[4] Nirmala B, Kumari G (2011). Foot drop after spinal anaesthesia: a rare complication. Indian J Anaesth.

[5] Makkar JK, Singh NP, Rastogi R (2015). Volume of contrast and selectivity for lumbar transforaminal epidural steroid injection. Pain Physician.

[6] Steverink JG, Piluso S, Malda J, Verlaan JJ (2021). Comparison of in vitro and in vivo toxicity of bupivacaine in musculoskeletal applications. Front Pain Res (Lausanne).

[7] Wang D, Vo NV, Sowa GA (2011). Bupivacaine decreases cell viability and matrix protein synthesis in an intervertebral disc organ model system. Spine J.

[8] Chee AV, Ren J, Lenart BA, Chen EY, Zhang Y, An HS (2014). Cytotoxicity of local anesthetics and nonionic contrast agents on bovine intervertebral disc cells cultured in a three-dimensional culture system. Spine J.

[9] Lee H, Sowa G, Vo N, Vadala G, O'Connell S, Studer R, Kang J (2010). Effect of bupivacaine on intervertebral disc cell viability. Spine J.

[10] Chang YW, Winkelstein BA (2011). Schwann cell proliferation and macrophage infiltration are evident at day 14 after painful cervical nerve root compression in the rat. J Neurotrauma.

